# Recent advances in mycobacterial transcription: insights beyond the general pathway

**DOI:** 10.1128/jb.00154-25

**Published:** 2025-06-24

**Authors:** Nilanjana Hazra, Jayanta Mukhopadhyay

**Affiliations:** 1 Department of Chemical Sciences, Bose Institute 30141, Kolkata, West Bengal, India; University of Notre Dame, Notre Dame, Indiana, USA

**Keywords:** RNA Polymerase, transcription, mycobacteria

## Abstract

The conventional idea of prokaryotic transcription represents a collection of pathways assembled from disparate studies across diverse bacterial species. This cumulative approach, though reveals core-conserved mechanisms, likely excludes the transcriptional pathways unique to an organism. The understanding of mycobacterial transcription suffers from such generalizations as its extreme GC bias, complex RNA polymerase (RNAP), abundance of short transcripts predominating its transcriptome, extensive σ factor utilization, and a constant battle against host stress implicates a distinct transcriptional landscape. This review highlights specific insights into mycobacterial RNAP architecture, promoter recognition, and elongation dynamics, against the general comprehensive narration of bacterial transcription.

## INTRODUCTION

RNA polymerase (RNAP) is central to the transcriptional machinery of a cell and plays an active role in regulating gene expression. It is a robust enzyme that initiates transcription, promotes elongation, and terminates the process in an ATP-independent manner. The catalytic core of RNAP, constituted by five subunits—two alpha (α), one beta (β), one beta prime (β′), and one omega (ω), is incapable of promoter-specific recognition of DNA (1, 2). An additional subunit σ facilitates promoter recognition by directly binding to the consensus sequence upstream to the transcriptional start site of DNA and directing the RNAP toward transcription initiation (3). Multiple subunits of RNAP act in joint concert to create a giant molecular machine that processes and assembles incoming nucleotide triphosphates (NTPs) into a growing RNA chain. The RNAP main channel acts as the “conveyor belt” and encompasses a passage primarily defined by the β and β′ subunits, which collectively orchestrate a series of conformational events upon downstream translocation of RNAP (4). The main channel allows DNA entry through the DNA duplex binding site (DBS), accommodates RNA-DNA complex at the hybrid binding site (HBS), and expels the nascent transcript through the RNA exit channel (5). The active centre is an integral part of the main channel, which provides the conformational space and necessary components for the catalytic reaction to generate a full-length productive RNA (4). The robustness, fidelity, and efficiency of transcription are intricately governed by the events focused at the active centre of RNAP.

Recent advances in structural and biophysical studies have led to an explosion in collective knowledge of the mechanisms involved in transcription. The prokaryotic transcription mechanism is mostly a generalization of extensive studies done on different bacteria across different genres of life. Most studies focus on the model organism, *Escherichia coli* (*Eco*), and some on *Thermus aquaticus (Taq*), *Thermus thermophilus (Tth*), *Bacillus subtilis (Bsu),* and *Mycobacterium* sp. (6). Although the fundamental principles of transcription initiation, elongation, and termination are elementally conserved across these organisms, unique adaptations to distinct environmental conditions may have led to significant structural, mechanical, and regulatory deviations in their RNAPs. A comprehensive attempt to understand the RNAP dynamics often omits subtle or essential variations in transcription pathways. For instance, the mycobacterial kinetic landscape of transcription initiation greatly varies from that of *Eco*. The *Mycobacterium tuberculosis* (*Mtb*) RNAP forms an unstable open promoter complex (RPo) but has a faster promoter escape rate than *Eco* (7). Additionally, the promoter consensus recognized by *Mtb* RNAP shows higher degeneracy than *Eco* RNAP, which accounts for high adaptability and robust gene expression, possibly to evade host immune responses in *Mtb* (8). Additional factors such as CarD and RbpA cooperatively stabilize the unstable mycobacterial RPo in promoters lacking a well-defined −35 element (9). This review compares and highlights the key differences in the mechanical and conformational events orchestrated primarily by *Eco* and *Mycobacterial* RNAP while summarizing the collective understanding of the general pathway of transcription.

## STRUCTURAL ORGANIZATION OF *ECO* AND *MYCOBACTERIAL* RNAP MAIN CHANNEL

**Fig 1:**
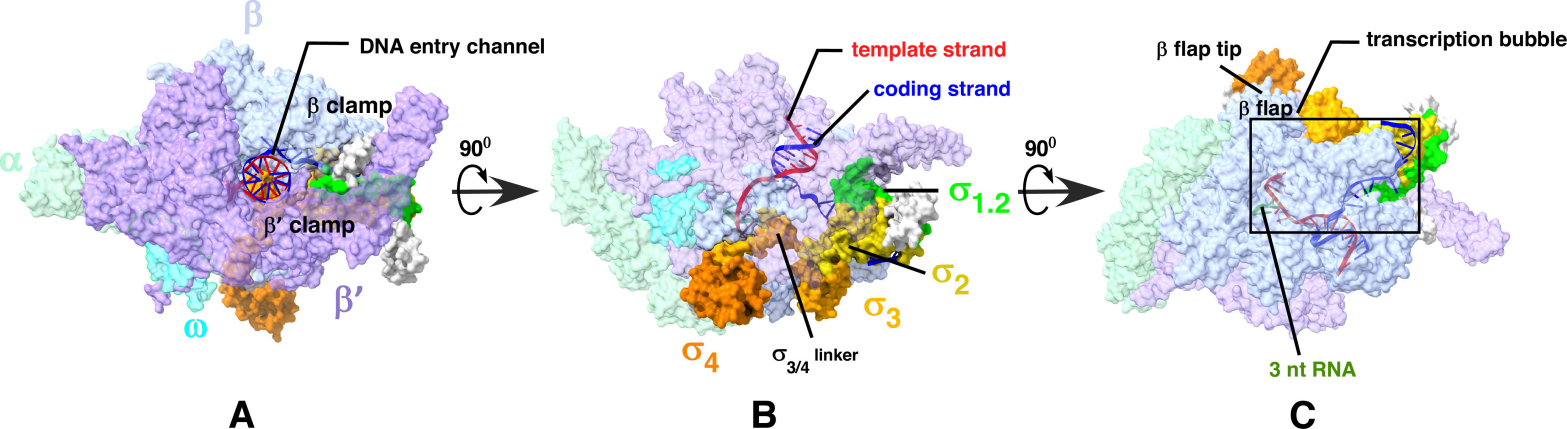
General structure of *Mtb* RNAP. Core subunits (αββ’ω) and σ subunit form the holoenzyme. The β, β′, and σ constitute the DNA entry channel (**A**), σ^A^ regions (σ_1.2_, σ_2_, σ_3_, and σ_4_) are distributed along the RNAP; σ-NTD with σ_1.2_ contacts the β and β′ clamp modules, σ_2_ contacts the coding strand to interact with the −10-promoter element, σ_3_ and σ_4_ regions contact the β-flap and β-flap tip of RNAP, respectively (**B and C**) [PDB 5UH5]. All 3D structural data have been represented using UCSF ChimeraX (17).

**Fig 2:**
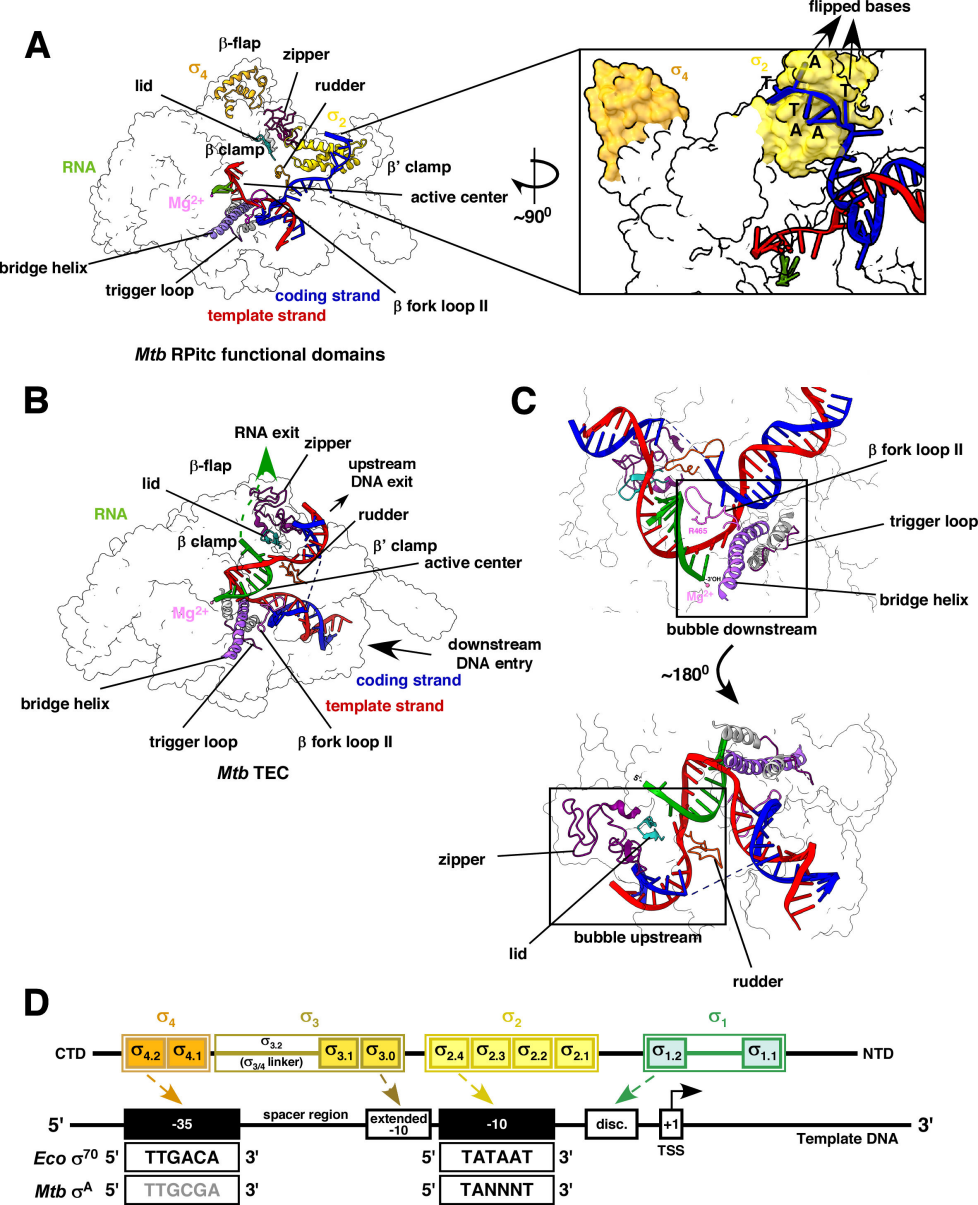
Structural representation of *Mtb* RNAP main channel components. (A) Structure of *Mtb* RNAP (RPitc with 3nt RNA) with key components (β fork loop II, bridge helix, trigger loop, rudder, zipper, lid, Mg^2+^ ion) contributing to *Mtb* transcription initiation [PDB 5UH5], oriented along the melted bubble. (B) Structure of *Mtb* RNAP (TEC) with 9 nt of RNA; key components oriented along the upstream and downstream transcription bubble [PDB 8E95]. (C) Upper: Downstream region of the transcription bubble and adjacent *Mtb* TEC functional components (β fork loop II, bridge helix or BH, and trigger loop or TL) oriented along the downstream bubble to process the incoming DNA. Lower: Upstream region of the transcription bubble and adjacent *Mtb* TEC functional components (rudder, zipper, and lid) oriented along the upstream bubble to process the RNA-DNA hybrid and the upstream DNA leaving the complex. (D) Schematic representation of housekeeping σ factor domains (1, 2, 3, and 4) and subdomains (1.1, 1.2, 2.1, 2.2, 2.3, 2.4, 3.0, 3.1, 3.2, 4.1, and 4.2) and major interactions with different promoter elements (−35 element, −10 element, extended −10 element, and discriminator element). The consensus sequences of the *Eco* core promoter—“TTGACA” (−35 element) and “TATAAT” (−10 element) are in black. The *Mtb* core promoter consensus sequences—“TTGCGA” (−35 element) is in gray to highlight a highly degenerate consensus, and “TANNNT” (−10 element) is in black (18).

**Fig 3:**
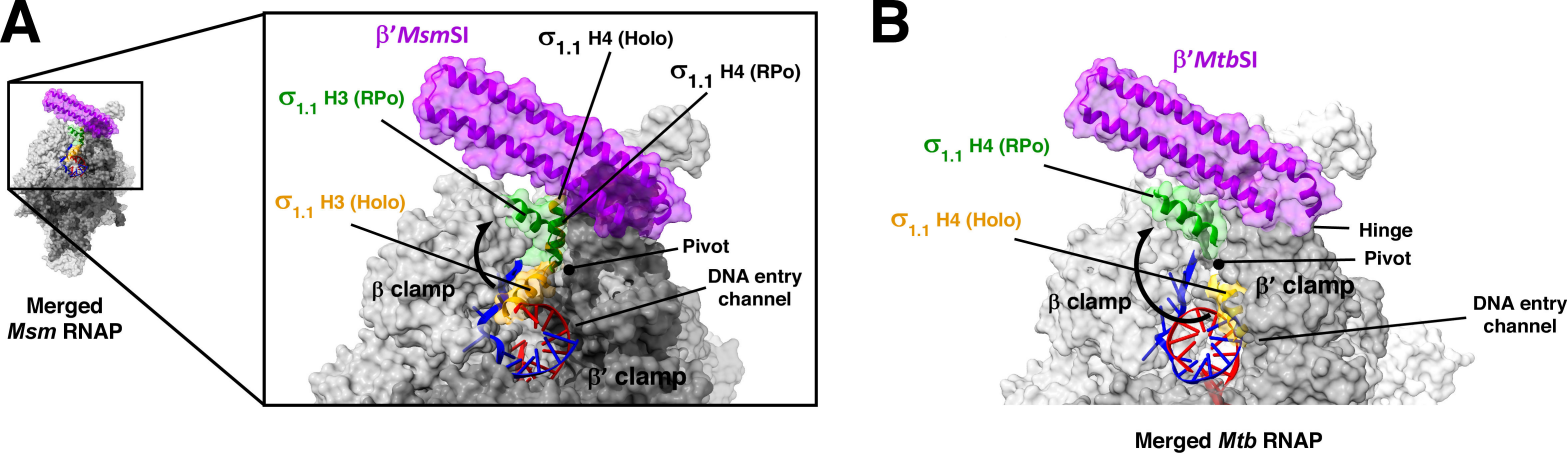
Mycobacterial β′SI and σ_1.1_ arrangement in holoenzyme and RPo. (A) Structural representation of *Msm* actinobacterial-specific insertion β′SI and σ_1.1_ helices (H3 and H4). The merged data from *Msm* holoenzyme (PDB 6EYD) and *Msm* RPo (PDB 5VI5) suggests σ_1.1_ H3 undergoes a ~90° rotation along the pivot from the DNA entry channel (in holoenzyme; yellow) toward the β’SI domain (in RPo; green), while σ_1.1_ H4 undergoes no substantial change upon open complex formation. (B) Structural representation of *Mtb* RPo with β′SI insertion and σ_1.1_ helices (H3 and H4). The merged data from *Eco* holoenzyme (PDB 4LK1) (19) and *Mtb* RPo (PDB 5UH5) suggest σ_1.1_ H4 undergoes rotation along the pivot from the DNA entry channel (holoenzyme; yellow) toward the β*′Mtb*SI domain (RPo; green) to vacate the channel for downstream entry of DNA (20).

The internal backbone architecture of the main channel of RPo is constructed by structural alignment of the β and β′ subunits of RNAP (4). The *Eco* σ^70^ domains (σ_1_, σ_2_, σ_3_, and σ_4_) joined by flexible linkers are sequentially arranged along this backbone to facilitate recognition of the promoter elements of the incoming DNA (Fig. 1B) (10). Each of the σ domains is further divided into subdomains—σ_1.1_ and σ_1.2_ constitute domain 1; σ_2.1_, σ_2.2_, σ_2.3_, and σ_2.4_ constitute domain 2; σ_3.0_, σ_3.1_, and σ_3.2_ (σ_3/4_ linker) constitute domain 3; σ_4.1_ and σ_4.2_ constitute domain 4 (Fig. 2D). In the absence of DNA, the *Eco* σ_1.1_ domain protects the active-center cleft of the RNAP holoenzyme and is displaced upon RPo formation (11). The displaced σ_1.1_ positions outside the active-center and interacts with the β′ clamp (12). Contrastingly, the N-terminal σ_1.1_ region in *Mycobacterium smegmatis* (*Msm*) is intrinsically disordered and is predicted to have a larger hydrodynamic volume, making it less likely to fit into the RNAP active site even without DNA (13, 14). In RNAP holoenzyme, the *Msm* σ_1.1_ is positioned outside the active-cleft and is strategically wedged between β and β′ clamps to restrict the entry of DNA into the active-site and regulate the formation of RPo (15). This domain must be rearranged to successfully accommodate the incoming DNA into the active center cleft of RNAP (Fig. 3A) (16).

The *Eco* σ_2_ domain at the active-center cleft primarily interacts with the β′ clamp and directs the events at the −10-promoter element, including DNA bending and melting to form the transcription bubble. The mycobacterial σ_2_ domain requires additional components such as CarD and RbpA to assist in initial −10 upstream element melting and bending to position the DNA toward the active site (9). The σ_3_ domain predominantly aligns above the β flap region positioned upstream to the active-center. The σ_3/4_ linker domain is wedged within the β′ clamp and the β-flap region to protrude into the RNA exit channel (Fig. 1B and C) (21–24). The σ_4_ is positioned far upstream at the outer edge of the β-flap tip (Fig. 1C) (25).

**Fig 4:**
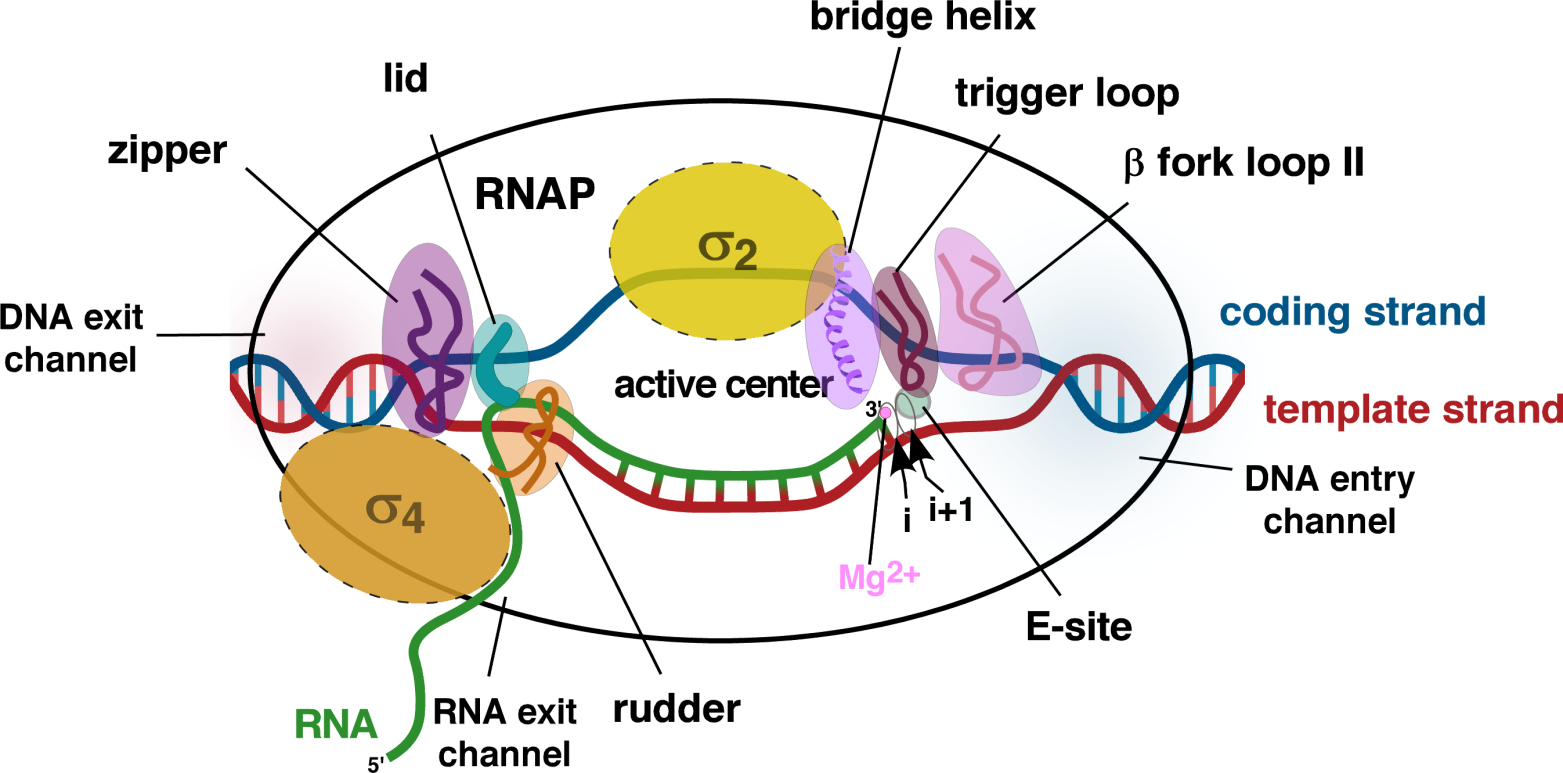
Schematic representation of RNAP main channel components. Double-stranded downstream DNA enters the RNAP through the DNA entry channel; the β fork loop II domain at the juncture of the DNA entry channel assists strand separation; the adjacent trigger loop docks the incoming NTP from the E-site at *i +* 1 position; upstream of *i +* 1 site, at *i* site, an RNA base with 3′-OH end, pairs with a DNA base on the template strand. The σ region 2 at RPitc interacts with the -10-promoter element on coding or non-template strand inside the melted bubble. At the upstream end of the bubble, β′ lid and rudder domains unwind the RNA-DNA hybrid and prevent reassociation, respectively. The RNA exits the RNAP through the RNA exit channel. The β′ zipper reanneals the template and coding strands, and the double-stranded upstream DNA exits the RNAP.

Five distinct channels facilitate the entry and positioning of the incoming and outgoing components to and from the RNAP. The downstream-duplex DNA channel (DNA entry channel) formed through the tip of the β and β′ clamp allows the dsDNA to enter the main channel (Fig. 1A and 4) (23). A 10 Å-wide “non-template strand” channel is formed by the σ_1.2_, σ_2_, and β clamp to position the non-template/coding strand away from the template strand in the transcription bubble (24). The “template-strand channel” is formed by σ_2_, σ_3_, β clamp, and β-flap regions, which position the template strand inside the active-center cleft for RNA catalysis (26). The secondary channel opens into the RNAP active-center to facilitate the entry of NTPs for utilization during RNA synthesis (27). Lastly, the 5′ end of the elongating nascent transcript exits the RNAP through the RNA exit channel at the upstream end of the main channel (Fig. 4) (28).

## DNA UNWINDING AND FORMATION OF OPEN PROMOTER COMPLEX

An RNAP-promoter closed complex (RPc) is formed when RNAP encounters its cognate promoter while “sliding” along the DNA or by effective collision (29, 30). *Eco* RNAP spans from around −56 upstream (UP element region or UPE) to +21 downstream of the transcription start site (TSS) on the promoter to unwind the dsDNA and form an open complex or RPo (31). The A/T-rich UP elements in *Eco* promoters are rare for *Mtb* promoters as its GC-rich genome disfavors the occurrence of such upstream motifs (13). The −35 element is indispensable for *Eco* RNAP promoter activity, while *Mtb* RNAP promoters often allow for high sequence degeneracy or lack a distinct −35 promoter element altogether (8). Furthermore, the −10 element of *Mtb* promoters exhibits high degeneracy (TANNNT), while the *Eco* −10 element shows less degeneracy and exhibits a well-defined consensus sequence (TATAAT) across most genes (18, 32, 33). Specific σ regions contact the promoter—the σ_2_ and σ_4_ domains bind the −10 element and −35 element, respectively, and σ_3_ contacts the extended −10 element (6, 34–38). The σ_1.2_ contacts a variable discriminator element of 5–8 nucleotides, located between the downstream end of the −10 element and the TSS (+1 site) of the non-template strand, influencing the stability of the RPo (39–41). This interaction influences the TSS selection. Disruption of σ_1.2_ interaction with the discriminator element or changes in its sequence can shift the TSS by ~3 base pairs (42). The negatively charged *Eco* σ_1.1_ domain is retained at the active-center cleft, blocking the DNA entry into the catalytic core of RNAP (11, 43). At this stage, RNAP binding to DNA is reversible and can disassemble from the promoter if RPo formation is energetically disfavored.

A series of events leads to the formation of a stable RPo. The displacement of *Eco* σ_1.1_ from the RNAP active site and the mycobacterial σ_1.1_ from the “gate” of the dsDNA entry of the active site unblocks the DNA entry channel (20, 44). An initial unstable transcription bubble is formed by melting approximately 13 bases (−11 to +2 position) of the dsDNA (45–47). The σ_2_ contacts the −10 promoter element to flip two bases at the −11 and −7 positions of the non-template strand toward its protein pocket, which stabilizes the melted conformation (Fig. 2A) (48).

**Fig 5:**
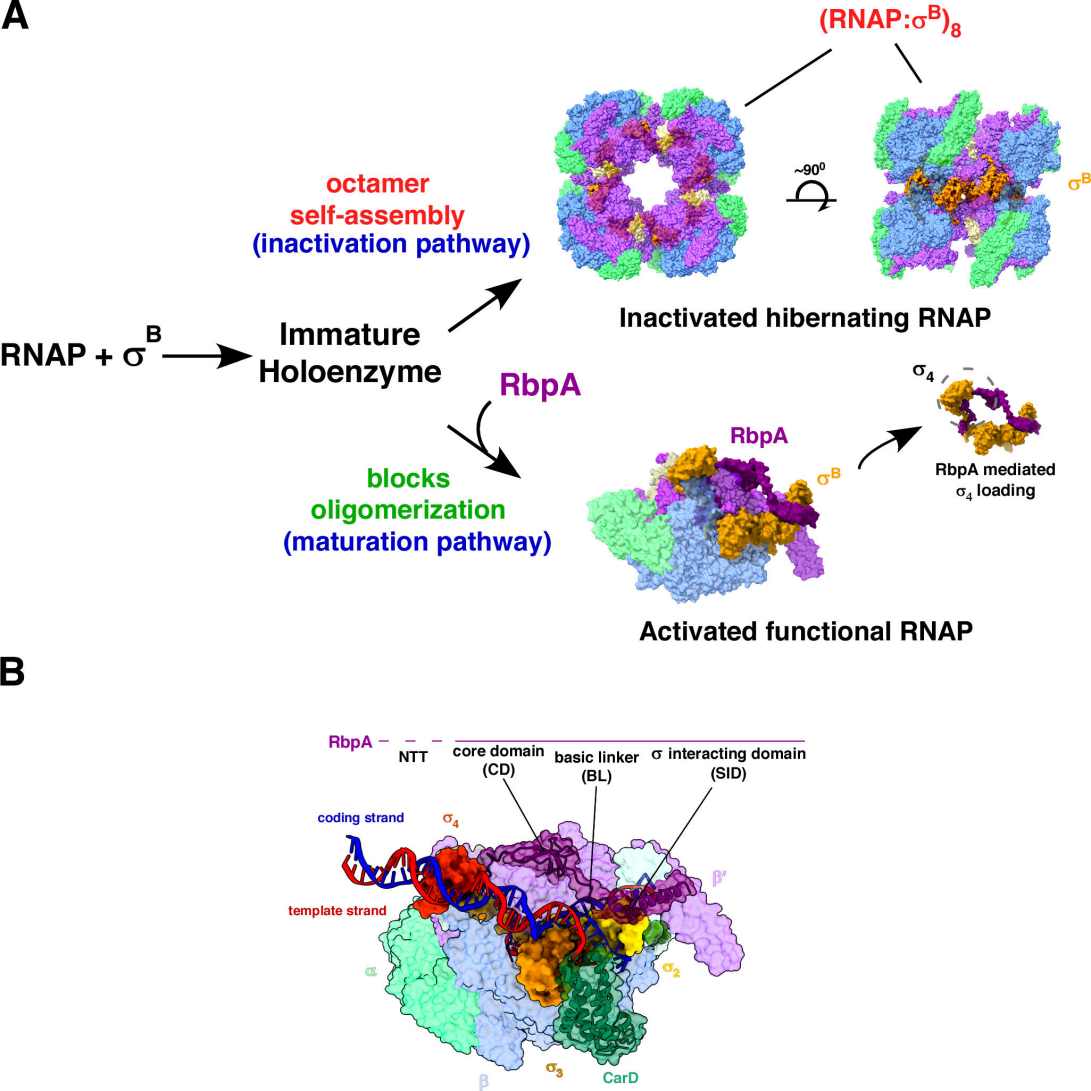
Additional pathways and components for *Mtb* RNAP regulation. (A) RNAP oligomerization pathway: RNAP interacts with σ^B^ to form an immature holoenzyme structure with an unloaded conformation of σ region 4 and self-assembles into an octameric inactive state. In the presence of RbpA, the immature holoenzyme enters the maturation pathway as RbpA blocks the self-assembly by σ_4_ loading onto RNAP [PDB 794U] (51). (B) Structural representation of *Mtb* RNAP (RPo) with additional factors—RbpA (with distinct domains—CD, BL, and SID) and CarD, assisting the open complex formation [PDB 6EDT] (52).

Unlike *Eco* RNAP, mycobacterial RNAP has to undergo complex conformational states to transition from initial reversible binding to a stable, irreversible RPo formation. Most mycobacterial promoters lack a well-defined −35 element, and the absence of interaction of σ_4_ with this promoter element limits RPo isomerization (8). The presence of −10 upstream interactions provided by extended σ_3_/−10 extended motif or the additional interactions of the RbpA protein with the −13 position facilitates the necessary anchorage to promote DNA bending toward the active-site cleft and positioning of the −10 element such that σ_2_ can effectively capture the flipped A nucleotide at position −11 (9, 34, 49, 50). Another protein, CarD, works synergistically with RbpA to enhance and stabilize the RPo formation by wedging a conserved tryptophan residue into the minor groove at the upstream edge of the −10 region, facilitating the initial opening of the DNA and preventing the collapse of the transcription bubble (9). Recent structural studies reveal an additional pathway unique to *Mtb* transcription where RNAP, in the presence of σ^B^, enters an inactivated hibernating state by forming octamers (Fig. 5A) (51). The σ^B^ region 4 remains unloaded in holoenzyme, conferring an immature state to RNAP, promoting oligomerization. In this unloaded state, the tip of the β-flap domain is rotated 111° toward σ_2_, adopting a “closed flap” conformation. This prevents the proper positioning of σ_4_ required to bind the −35 element and interact with the β-flap to define the RNA exit channel. This subsequently traps the RNAP into an inactive conformation prone to octameric oligomerization. The repressed transcriptional state of RNAP is rescued upon the addition of RbpA, which induces allosteric conformational changes to RNAP, leading to σ_4_ loading to the RNA exit channel and subsequent RNAP maturation. Such oligomeric transcriptional repression is unprecedented or unreported for any other organism.

Several models describe the probable DNA unwinding process during RPo formation in the context of *Eco* RNAP clamp movements. The clamp-opening model states that the β′-clamp swings out to expose the active site. The dsDNA is loaded onto the cleft and melted in sequential order (53–55). The clamp collapses back to lock the melted DNA inside the active-center cleft. Contrastingly, the external-unwinding model proposes that DNA melts outside the active site and enters the site in a single-stranded form without requiring β′ clamp opening or closing (56, 57). Recent structural evidence supports that *Mtb* housekeeping RNAP follows the clamp-opening model for promoter melting (52).

The *Mtb* β′ subunit harbors an additional ~100 residue sequence insertion (β*′ Mtb*SI) of Actinobacterial lineage, which folds as a ~70 Å long α-helical coiled-coil structure that spans from the β′ clamp to extend toward the RNAP active-center (Fig. 3). Among the two anti-parallel α helices, one of the α helices faces the RNAP active-center and exhibits a net positive surface charge, and the other with a net negative surface charge, spans outward (13). This β*′ Mtb*SI domain functions as a “gate” to lock the DNA inside the active-center and must displace around the “hinge” region at the β′ clamp-β*′ Mtb*SI juncture to allow DNA entry into the active-center (Fig. 3B) (20). A deletion of β’*Mtb*SI domain reduces the *Mtb* RPo stability by 10-fold. Additionally, the *Mtb* σ_1.1_ orients itself between the β*′ Mtb*SI and dsDNA and assists β*′ Mtb*SI in trapping the DNA into the active-center cleft (Fig. 3B). A joint deletion of β*′ Mtb*SI and σ_1.1_ leads to a 70-fold defect in RPo stability in *Mtb* (20). Contrastingly, *Eco* RNAP lacks β’*Mtb*SI naturally, and its σ_1.1_ deletion does not affect RPo stability, indicating an absence of the dsDNA “locking” mechanism unique to *Mtb* RNAP.

## INITIAL TRANSCRIBING COMPLEX (RPITC) FORMATION AND PROMOTER ESCAPE

RPo is stabilized by initial incorporations of the first two NTPs at +1 and +2 positions (58). This initial NTP addition gives rise to the RNAP-DNA-RNA hybrid or RPitc (Fig. 2A). *Mtb* RNAP exhibits a slower initial NTP incorporation rate than *Eco,* which is not improved by CarD or RbpA interactions (7). As the RPitc attempts to translocate, it pulls in more DNA and adds NTPs to the growing RNA chain. However, it fails to escape the promoter to release a short stretch of an abortive transcript as the elongating RNA sterically clashes with the σ_3.2_ (σ_3/4_ linker domain) that blocks the RNA exit channel (24, 59). RNAP then enters a cyclic phase of short RNA synthesis (~2 to 13 nucleotides), followed by release, and then reverting to the initial open complex (RPo) configuration to start the process in repeat (60–62). As a result, excess downstream DNA is pulled into the active site without upstream dislocation of the −35-promoter element, leading to a scrunched or coiled conformation of the melted DNA (63). An enlarged and distorted transcription bubble of ~25 bases is formed, accumulating potential energy like a compressed spring. Upon reaching a critical threshold, it provides enough kinetic energy to RNAP to shoot downstream and finally escape the promoter (64). The promoter escape also displaces the σ_3/4_ linker domain to unblock the RNA exit channel, severs the σ_4_ contacts, and promotes productive RNA synthesis (24, 65, 66).

Apart from RNAP architecture, promoter escape kinetics are also affected by the strength of the promoter. Surprisingly, for *Eco* RNAP, the efficiency of promoter escape is negatively correlated to promoter strength. Strong promoters exhibit higher yields of abortive transcripts, leading to weakened promoter escape efficiency (59, 67, 68). A stable association with the promoter disfavors the energetics required to break the initial contact of RNAP and DNA to promote escape. Additionally, the nature of the initial transcribing sequences (ITS) also determines the stability of the DNA-RNA hybrid inside the active site, affecting the promoter escape efficiency (69). *Mtb* RNAP exhibits faster overall promoter escape kinetics despite having a slower initial NTP incorporation rate than *Eco* (7). This suggests that the transition from RPitc to the elongation complex (RDe or TEC) occurs more efficiently in *Mtb* than in *Eco* after the initial NTP addition to form the RNA transcript. Such differences in promoter escape kinetics can be attributed to the structural and conformational complexity of mycobacterial RNAP and the unconventional architecture of mycobacterial promoters. The factors CarD and RbpA slow down the overall promoter escape process by stabilizing RPo and modulating the transcription initiation dynamics in *Mtb* (Fig. 5B). Finally, a successful promoter escape is proposed to facilitate σ-release from *Eco* RNAP (70). However, recent FRET-based studies and single-molecule experiments challenge a model of obligate σ release, suggesting some σ retention on transcription elongation complexes (TECs) (22, 71, 72). The half-life of σ-retention was estimated to be ~50 s, sufficient for RNAP to transcribe past ~1 kb of DNA (73, 74). Additional studies in *E. coli* suggest that σ factor retention facilitates σ-dependent pausing events during mature elongation at downstream promoter-like motifs (70, 75).

## TRANSCRIPTION ELONGATION, ACTIVE-CENTER DYNAMICS, AND RNA EXIT

**Fig 6:**
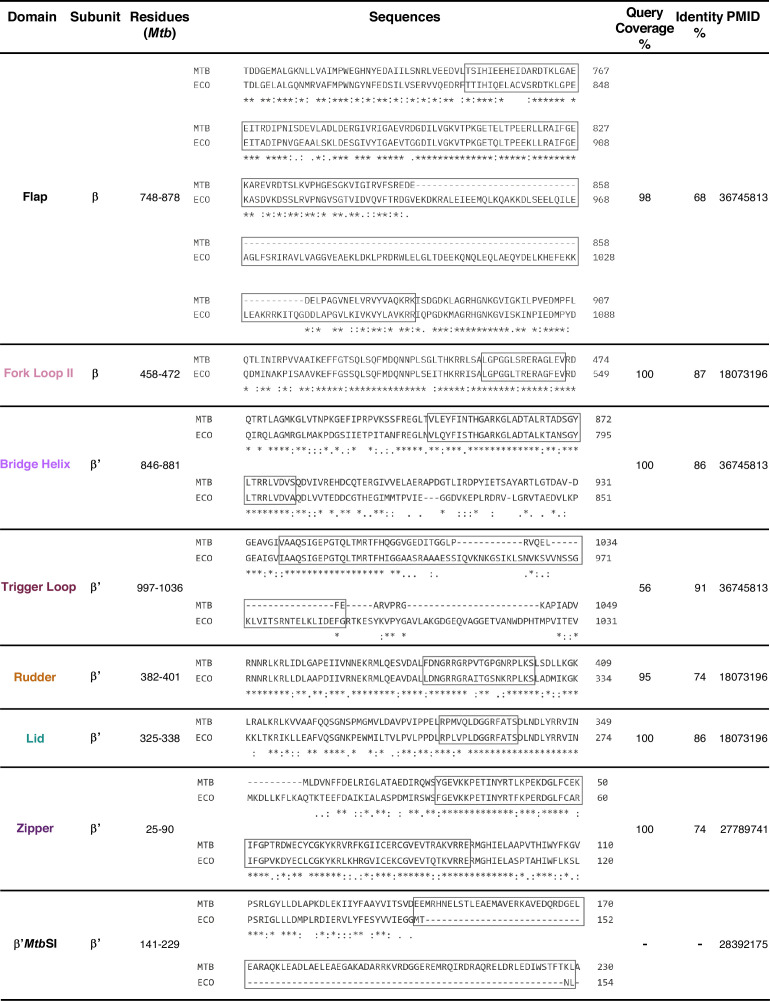
Sequence information of *Mtb* RNAP main channel components. Tabular representation of sequence information (residues, sequence alignment with *E. coli* RNAP, query coverage %, and identity % of aligned residues) of *Mtb* RNAP main channel components from Fig. 4. The query coverage (%) indicates the percentage of the *Mtb* sequence covered by the *Eco* sequence in the alignment. Domain names are color coordinated according to Fig. 4 schematic data; domain sequences are represented inside the boxes. The *Mtb* RNAP or *Eco* RNAP domain sequences are derived from PMIDs 36745813 (78), 18073196 (79), 27789741 (80), and 28392175 (20).

RNAP escapes the promoter to transition into the elongation phase. The elongating RNAP (TEC) undergoes a series of synchronized conformational changes to grow the RNA chain one base at a time. Several key components, greatly conserved in bacteria, constitute the active-center to work in joint concert during transcription elongation (Fig. 4 and 6). The RNAP main channel or active center is mainly constructed by β′ clamp, β clamp, and the β flap domains (Fig. 2) (76). The β and β′ subunits give rise to a “crab-claw” structure to jointly orchestrate the opening and closing of the RNAP to process the DNA inside the active center (4). The dsDNA enters RNAP through the downstream DNA entry channel formed by the β′ clamp and β clamp domains (Fig. 2A and B). Antibiotics that target the β subunit of *Eco* RNAP usually bind to lock the clamp at a fixed conformation (open or closed), disrupting its mechanical freedom (77). Abrogation of the conformational dynamics of RNAP renders it inactive to perform transcription.

The β Fork Loop-II is a component of the β subunit located at the downstream end of the active-center and is highly conserved across bacteria (Fig. 2 and 4) (81). It is positioned at the junction of the DNA entry channel to assist the strand separation of the downstream dsDNA entering the RNAP (79, 82). The arginine residue of the F-Loop II domain (*Eco* R540, *Mtb* R465) is indispensable for unwinding the DNA, which, if mutated, severs the catalytic activity of RNAP (Fig. 2C) (83). Upon DNA unwinding, the β fork loop-II ensures the correct positioning of the unwound DNA for subsequent NTP addition (82). An entry site or E-site positioned inside the secondary channel of RNAP facilitates NTP entry and binding to the active center (Fig. 4) (84–86). An important functional component contributed by the β′ subunit of RNAP is located adjacent to the β fork loop-II, inside the active site, called the trigger loop (Fig. 2 and 4) (79). Specific conformational changes of the trigger loop assist NTP incorporation to the elongating RNA and the proofreading activity of RNAP, ensuring fidelity of the transcription process (87). E-site and trigger loop function together as the incoming NTPs are initially docked into the E-site, and the trigger loop ensures the loading of the correct NTP to the 3′-OH of the RNA (Fig. 4) (82). The β′-subunit of RNAP constitutes a functional α helix domain called bridge helix located at the active site, adjacent to the trigger loop (Fig. 2C and 4) (88). It assists the translocation of the RNA-DNA hybrid, ensuring optimal alignment of the 3′ end of the RNA for nucleotide addition (89, 90). It oscillates between an α-helical and an unfolded conformation to initiate an SN2 nucleophilic attack during the catalysis of RNA (91–93). As the RNA synthesis begins, the 3′ OH end of the RNA is positioned at the *i*-site, and the next NTP is placed at the *i +* 1 site (Fig. 4) (94). The 3′ OH group of the phosphodiester backbone of the RNA initiates an SN2 nucleophilic attack on the α-phosphate of the NTP facilitated by Mg^2+^ ion (95–97). After the bond formation, one molecule of PPi (pyrophosphate) is released to drive the necessary conformational changes for RNAP translocation. The RNAP then unwinds one base down the template to position the newly formed 3′OH end of the RNA at the *i*-site.

The DNA rewinds at the upstream end to maintain a constant size of the transcription bubble. A structural component of the β subunit called the β-zipper facilitates re-annealing of the template and non-template DNA, upstream of the transcription bubble (Fig. 2C and 4) (98). This retains the structural integrity of the DNA exiting the RNAP. A sub-domain of the β subunit called β flap occupies the active center and constructs the upstream end of the active center that covers the RNA exit channel (Fig. 2A and B). This domain interacts with σ_3_ and σ_4_ domains, inside and outside the active center, respectively (25, 99, 100). The lid domain of β′ subunit is located at the upstream end of the active center cleft and stacks upon the upstream base pair to unwind the RNA-DNA (Fig. 2C and 4) (4, 101, 102). The β′ rudder interacts with the single-stranded unwound DNA from the RNA-DNA hybrid to prevent reassociation of the nascent RNA to the upstream DNA (Fig. 2C and 4) (103–105). The β-zipper finally re-anneals the template and non-template strands of the upstream DNA as the nascent RNA moves along the main channel to exit through the RNA exit channel and leave the active center (Fig. 2B and C) (98).

**Fig 7:**
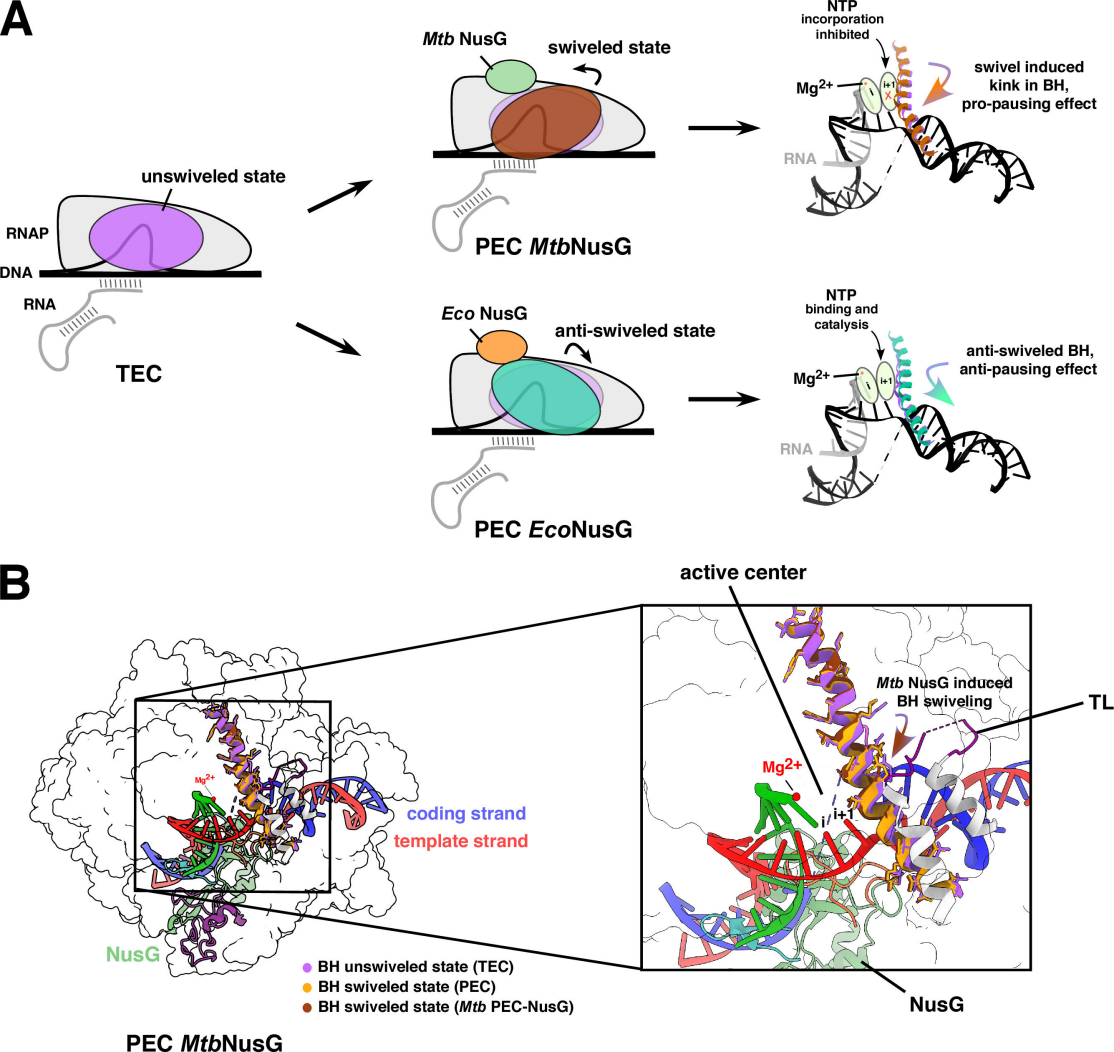
*Mtb*NusG induces pro-pausing by RNAP swiveling. (A) Schematic representation of NusG-mediated swiveling or anti-swiveling effect on *Mtb* and *Eco* PEC (paused elongation complexes). Pro-pausing *Mtb*NusG induces swiveling of bridge helix (BH) towards the *i* + 1 site blocking the NTP incorporation to the elongating 3′-OH end of the RNA transcript. Contrastingly, *Eco*NusG promotes anti-pausing by negative swiveling of BH away from the *i* + 1 site to allow NTP binding and elongation. (B) Structural representation of *Mtb* PEC (merged), pro-paused due to BH swivelled state induced by *Mtb*NusG. The initial position of BH (0^0^) in TEC (purple; PDB 8E95) swivels toward the *i* + 1 site (3.13^0^) promoting pausing (PEC; orange; PDB 8E8M); the presence of *Mtb*NusG swivels the BH deeper into the *i* + 1 site (3.44^0^) promoting pro-pausing (brown; PDB 8E74) of the RNAP (106).

RNAP encounters specific signals to halt its translocation along the downstream DNA during elongation (106). The sequence of the DNA, the formation of RNA secondary structures, and the presence of transcriptional regulators can cause RNAP to stall mid-transcription (78, 107–109). Slow transcription rates, structural and conformational rearrangements of the TEC, and specific interactions with the DNA or the nascent RNA influence pausing events (110). These off-pathway events during elongation allow for a strategic response against cellular stress by altering the gene expression pattern. RNAP enters a temporary state of an off-pathway, short-lived pause state called elemental pause (111). Post NTP incorporation, RNAP can isomerize into a catalytically inactive state (elemental paused TEC or ePEC) to hinder the completion of the nucleotide addition cycle (NAC) (112). Specific sequence interactions of RNAP to the DNA elicit elemental pauses; for instance, a sudden drastic variation in the AT/GC content affects the RNA-DNA hybrid, causing the elongating RNAP to pause and prevent misincorporation (113, 114). Brief elemental pauses can convert to long-term pauses due to RNAP backtracking events on specific DNA sequences, such as long U-rich fragments or other sequence features contributing to weak RNA-DNA hybrids (115–118). During an NTP misincorporation, the paused RNAP can move backward on the template DNA to disengage the 3′ end of the RNA, extruding it into the secondary channel (119). Backtracking stabilizes the paused RNAP (Backtracked elongation complex or BEC), ensures transcriptional fidelity, and allows the recruitment of essential elongation factors (such as GreA and GreB) to rescue RNAP and resume transcription (115, 120, 121). The formation of a stable RNA-hairpin structure in the RNA exit channel can pause the elongating RNAP (108). Specific sequences in the transcribed RNA promote secondary structure formation by inducing direct or allosteric conformational changes on RNAP to render it inactive. The β-flap tip helix (FTH) domain repositions in response to RNA-hairpin formation, impairing the trigger loop (TL) folding dynamics and halting catalysis. An essential elongation factor, NusA, binds *Eco* RNAP and nascent RNA to stabilize and prolong the pausing event. NusA is expected to have similar functional roles in mycobacteria as both *Mtb* and *Eco* NusA bind the *rrn nut* sites (N-utilization sites found in ribosomal RNA operons) and destabilize RNA secondary structures (122). *Mtb* RNAP exhibits a greater propensity for pausing than *Eco* RNAP (123). In the presence of an elongation factor NusG, and upon encountering pause signals, *Mtb* RNAP swivels and enters a half-translocated paused state (Fig. 7) (106). The *Mtb* NusG stabilizes this swiveled state by orienting the bridge helix further toward the *i +* 1 site to block NTP incorporation and exert a pro-pausing effect on the RNAP (Fig. 7A and B). Contrastingly, *Eco* NusG suppresses the swiveling conformation and facilitates RNAP translocation to confer an anti-pausing effect (Fig. 7A) (78, 106).

## RNAP UNLOADING AND TRANSCRIPTION TERMINATION

**Fig 8:**
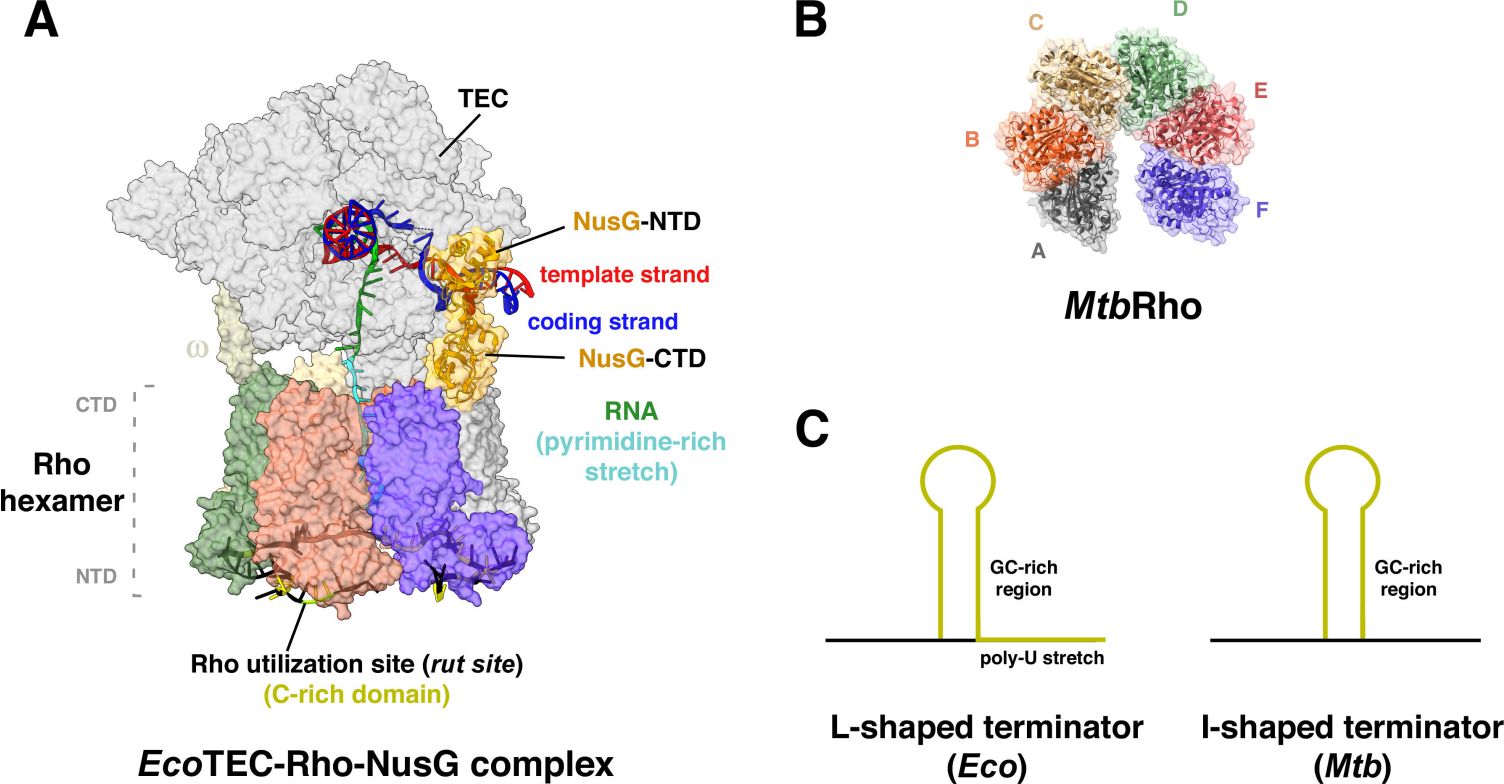
Mechanisms of transcription termination. (A) *Eco* termination complex with Rho factor. RNA extruding from TEC is trapped by Rho (pyrimidine-rich stretch); RNA with *rut* sites (C-rich) is aligned along the surface of Rho-NTD. NusG connects TEC-Rho and aids the formation of closed-ring active state of Rho hexamer (PDB 8E6X and 8E6W) (128). (B) Open ring structure of *Mtb*Rho factor with hexameric units aligned adjacent to each other (A–F) (PDB 7OQH). (C) Predominant shapes of intrinsic terminators—“L-shaped” terminators in *Eco*, “I-shaped” terminators in *Mtb*.

RNAP encounters termination signals to unload the complex from DNA, release the mature RNA, and effectively conclude the transcription process. Transcription termination in bacteria can be achieved by two pathways—Rho-dependent and intrinsic termination (124, 125). Rho-dependent termination involves a hexameric factor, Rho, that recognizes and binds to a C-rich domain followed by a pyrimidine-rich stretch on the emerging RNA from the TECs (Fig. 8A) (126, 127). The Rho hexamer initially forms a catalytically inactive open-ring structure, which upon interaction with an elongation factor, NusG, transitions into a closed-ring active state (128). After the formation of the closed ring, Rho translocates on the elongating RNA toward the moving RNAP in an ATP-dependent manner. NusG bridges the gap between Rho and RNAP and aligns the Rho hexamer relative to RNAP to prevent futile cycles of rotation during ATP hydrolysis (Fig. 8A) (128). Finally, the Rho-factor exerts mechanical force to disrupt the TECs from the DNA to promote termination.

Both intrinsic and Rho-dependent termination have been demonstrated in mycobacteria; however, Rho-dependent termination has emerged as the dominant mechanism over the intrinsic pathway (129). Only 2%–4% of all termination sites (TTS) in *Mtb* are found to be canonical (stem-loop structures followed by a poly-U stretch) terminators, while approximately 54% of all TTS and 60% of conditional TTS (regulated in response to physiological signals) are found to be Rho-dependent (130). Mycobacterial Rho factor is essential for viability, which, if downregulated, leads to transcriptional readthrough, accumulation of pervasive transcripts, and eventual cell death (131). Unlike *Eco*, *Mtb* Rho factor recognizes purine-rich sequences and exhibits lower susceptibility towards bicyclomycin (BCM) (132).

The Rho-independent or intrinsic termination depends on specific GC-rich signals on DNA, which, upon RNA formation, generate a secondary hairpin structure (Fig. 8C) (124). These structures are followed by a poly-U stretch that slows down, stalls, and dismantles the RNAP from the DNA to terminate the transcription process and release the mature RNA (133). The intrinsic terminators in mycobacteria are scarce and differ considerably from *Eco* terminators. *Eco* terminators typically comprise a polyU stretch that follows a hairpin structure formed by a GC-rich palindromic region in the RNA transcript, giving rise to an “L-shaped” structure (Fig. 8C). The poly-U stretch enhances termination efficiency as AU base pairing between the RNA-DNA hybrid allows for an easy destabilization of the complex, release of the nascent transcript, and effective termination. Contrastingly, mycobacterial genes predominantly utilize “I-shaped” terminators, which lack the polyU tail (Fig. 8C), located within 50 bp downstream of the stop codon, to efficiently terminate transcription right after encountering the end of the coding sequence (134, 135). In *E. coli*, the elongation factor, NusA stimulates termination at intrinsic terminators by interacting with the terminator hairpin and the RNA exit channel (136, 137). However, mycobacterial NusA does not enhance termination at canonical or non-canonical (lacking polyU tail) terminators (137). Additionally, unlike *Eco* NusG, mycobacterial NusG has a pro-pausing effect on RNAP and strongly stimulates intrinsic termination upon encountering suboptimal polyU tail at canonical terminators (137, 138).

## CONCLUDING REMARKS

The complex nature of RNAP equips mycobacteria to transcribe their GC-rich genome. Multiple pathways have deviated from the conventional mechanisms to provide transcriptional fitness to the mycobacterial RNAP. From unique sequence insertions to the ability to recognize diverse promoter motifs, mycobacterial RNAP has gained efficiency and robustness over *Eco* RNAP. With the support of transcriptional regulators such as CarD and RbpA, mycobacterial RNAP has overcome kinetic constraints during promoter unwinding and open complex formation (7). An exceptionally high ratio of alternative σ factors to genomic size has enabled mycobacteria to withstand stress (139–141). Most of these σ factors initiate transcription from various GC-rich promoter sequences (33). To demonstrate efficient termination, mycobacterial RNAP has adapted to utilize non-canonical (I-shaped) terminators that inherently lack polyU tails, a critical adaptation given that high GC content likely disfavors the formation of canonical (L-shaped) terminator structures (134, 135). Such remarkable survival strategies and adaptability of *Mtb* substantiate the presence of novel cellular processes, most of which converge at the point of transcriptional regulation. This necessitates scientific attempts to develop a dedicated mycobacterial transcriptional paradigm, reducing reliance on potentially incomplete or inaccurate extrapolations from distantly related bacterial systems.
